# Current efforts and challenges facing responses to 2019-nCoV in Africa

**DOI:** 10.1186/s41256-020-00148-1

**Published:** 2020-05-06

**Authors:** Don Eliseo Lucero-Prisno, Yusuff Adebayo Adebisi, Xu Lin

**Affiliations:** 1 Department of Global Health and Development, London School of Hygiene and Tropical Medicine, London, UK; 2 Faculty of Pharmacy, University of Ibadan, Ibadan, Nigeria; 3 Department of Thoracic Surgery, the First Affiliated Hospital, Zhejiang University School of Medicine, Hangzhou, 310003 P.R. China

**Keywords:** 2019-nCoV, SARS-CoV-2, COVID-19, Coronavirus, Africa, Outbreak, Global Health, Pandemic

## Abstract

The novel coronavirus is a pandemic that has started to creep into Africa thus making the virus a truly global, health security threat. The number of new 2019-nCoV cases has been rising in Africa, though currently lower than the cases reported outside the region. African countries have activated their Emergency Operations Centres to coordinate responses and preparedness activities to the pandemic. A series of measures such as restricting travel, case detection and contact tracing, mandatory quarantine, guidance and information to the public among other efforts are being implemented across Africa. However, the presence of porous borders, the double burden of communicable and non-communicable diseases, poverty, poor health literacy, infodemic and family clustering, and most of all, weak health systems, may make containment challenging. It is important for African countries to continue to intensify efforts and address the challenges to effectively respond to the uncertainty the pandemic poses.

## Background

The World Health Organization (WHO) declared the novel coronavirus (also called COVID-19, or 2019-nCoV, or SARS-CoV-2) outbreak as pandemic after it was previously referred to as a global health emergency. As coronavirus spreads across the globe, African countries are not spared from the impacts on the global health security. As of 18 April 2020, 21,761 cases and 1082 deaths have been reported in 52 African countries and the most affected countries (number of confirmed cases) are South Africa (3034), Egypt (3032), Morocco (2685), Algeria (2534) and Cameroon (1017) [1]. In the WHO African Region, as of 18 April 2020, 45 out of 47 member states have reported 2019-nCoV cases with no cases reported in Comoros and Lesotho [2].

The pandemic continues to evolve across Africa and the mortality rate is rising. As of 14 April 2020, fifteen countries (Burundi, Democratic Republic of Congo, Sudan, Algeria, Egpyt, Mauritania, Morocco, Angola, Botswana, Malawi, Zimbabwe, Cape Verde, the Gambia, Liberia and Mali) have reported case fatality rates higher than the global case fatality rate of 6% [1]. As of 11 March 2020, only Algeria, Cameroon and Nigeria had reported local transmission [3]. By 18 April 2020, more than 40 African countries have reported local transmission. This further reinforces the presence of human-to-human transmission of 2019-nCoV.

This article aims at providing a critical commentary on the current efforts against 2019-nCoV pandemic and the challenges facing its responses in the African continent.

## Current efforts

There has been a rapid response to the pandemic from Africa’s public health systems well before cases were reported. On 3 February 2020, the Africa Centres for Disease Control and Prevention established the Africa Task Force for Novel Coronavirus. The task force has been working with WHO African Region on community engagement, surveillance, including screening at points of entry, infection prevention and control in health-care facilities, clinical management of people with severe 2019-nCoV infection, laboratory diagnosis, and risk communication [4]. Moreover, with the previous programs addressing Ebola of affected regions in Africa, some of the systems implemented and lessons learned may have provided the groundwork in addressing the current pandemic.

African countries have activated the Emergency Operations Centres to coordinate response and preparedness activities. Training sessions have also been set up across the African continent to equip the rapid response team with knowledge and skills. Public health programs have been ramped up by national authorities. WHO African Region has also set up a public interactive dashboard for the visualization of the 2019-nCoV situation in the region. Furthermore, in preparation for a possible increase of cases, some African countries have increased their healthcare capacity. For instance, Morocco currently has 44 hospitals with 32 specialized centres that are fully equipped in response to the pandemic [5].

Nigeria, Algeria, Senegal, South Africa and many African countries have laboratories which conduct in-country testing for 2019-nCoV. Between 2 February and 18 April 2020, in the WHO African Region, the laboratory testing capacity has increased from two to forty-four countries [2]. Efforts are currently underway to increase diagnostic capacity across the continent. Many countries have identified isolation and quarantine centres. Effort is also ongoing to ensure effective contact tracing of potential contacts with infected persons.

Work on strengthening the surveillance system in Africa have also been started prior to the pandemic. Partners have also indicated their support to countries in the implementation of early investigation studies, such as the First Few X (FFX) case and contact investigation protocol for timely estimation of transmissibility and severity of 2019-nCoV. However, as of 18 March 2020, only fourteen countries have expressed interest in the implementation of FFX [6]. As of 8 April 2020, four countries (South Sudan, Madagascar, South Africa and Cote d’Ivoire) have started the implementation of early investigation protocol. Nigeria is the first African country to publish 2019-nCoV genomic data from index patient.

Airports across the continent are testing passengers’ temperatures upon arrival. Most African airlines have temporarily suspended flights to high-risk countries. As of 18 April 2020, in the WHO African Region, 35 countries have implemented total refusal of entry into their territories, 9 countries are implementing refusal of entry of passengers from high risk countries and 3 countries allow entry with 14 days quarantine upon arrival [2]. Most governments have temporarily closed educational institutions in order to limit the spread of the virus. Gatherings have been banned with police enforcement in the Gambia, Nigeria, Rwanda, Zimbabwe, South Africa, and Senegal among others. Curfews and interstate travel ban have been implemented in some countries.

In the area of clinical case management, efforts to develop and ensure evidence-based treatment guidelines and protocols for the management of 2019-nCoV are ongoing in Senegal, Nigeria, Algeria, South Africa, Madagascar and other countries across the continent.

Even though it is too early to assess the effectiveness of the responses, countries have adopted measures worth learning from; for instance, simplified triage strategies and proactive screening (Uganda), handwashing stations at transport hubs (Rwanda), WhatsApp chatbots providing reliable information, and rapid testing diagnostics (Senegal), and volunteer-staffed call centres via toll-free telephone lines and celebrity campaigns (Nigeria) [7]. Countries like São Tomé and Principe, Zambia, Nigeria, Burkina Faso have also been using telecommunication companies to support dissemination of information regarding prevention and control practices. Togo is leveraging on medical students to raise awareness among grassroot communities while Senegal has mobilized religious leaders. With the support of donors and governments, some African countries are also making efforts to provide relief materials, food items and other supports to the poor.

## Challenges

The Johns Hopkins Center for Health Security publication reported African continent is least prepared to respond to health emergencies, treat the sick and protect health care workers [8]. The weak healthcare system and high prevalence of malnutrition, malaria, HIV/AIDS, and tuberculosis are further challenges. Additionally, African countries are experiencing a double burden of diseases [9]. Even though the continent has relatively lower cases, efforts need to be augmented to ensure that spread is placed under control. Some global health experts have attributed this to low testing rates and its young population. There is a need to augment efforts in ensuring widespread in-country testing which has been a major challenge. Development of cost-effective, rapid and accurate diagnostic tests is much-needed to ensure early detection of cases.

Africa has the lowest capacity to provide critical and intensive care in the world [10]. Severe 2019-nCoV cases may lead to respiratory insufficiency syndrome which require ventilation support. For successful treatment of this complication, it is dependent on the availability of ventilators, power supply, and oxygen, which is a challenge. Some countries also have challenges in setting up conducive isolation centers which may further discourage or halt mandatory quarantine of suspected cases.

Social and religious gatherings are not uncommon in Africa. Many countries have banned such gatherings and this has received resistance from some people across the region thus affecting physical distancing. Family clustering, poor health literacy, infodemic and poverty may also contribute to a poor response. The lockdowns and stay-at-home strategy have halted business activities making the poor the most vulnerable to the economic consequences, posing further challenges to adhere to precautionary measures. Price hikes for masks and hand sanitizers have also been reported. The presence of porous borders and congested cities and slums are also some of the issues facing some African countries. Bans on international travel may also constitute obstacles for supporting response and preparedness operations. This further reinforces the need for unique approaches to ensure effective responses to the pandemic in African continent.

## Conclusion

Most African countries have set up measures to respond to the 2019-nCoV pandemic. However, these efforts are not without challenges. It is important for Africa to continue to intensify efforts and address the challenges to effectively respond to the uncertainty the pandemic poses. This also calls for a unique approach to ensure a proactive response. Early isolation, effective knowledge sharing, contact tracing, good reporting and surveillance system, parallel testing, collaborations and cooperation within and outside the continent, sustained community engagement and diseases prevention and control measures, including physical distancing and proper hand and respiratory hygiene should continue to be prioritised in the region.

## Data Availability

Not applicable.

## Abbreviations

2019-nCoV, or COVID-19, or SARS-CoV-2: Novel coronavirus
WHO: World Health Organization
FFX: First Few X
